# Effectiveness of school-based preventive chemotherapy strategies for sustaining the control of schistosomiasis in Côte d’Ivoire: Results of a 5-year cluster randomized trial

**DOI:** 10.1371/journal.pntd.0008845

**Published:** 2021-01-15

**Authors:** Mamadou Ouattara, Nana R. Diakité, Patrick K. Yao, Jasmina Saric, Jean T. Coulibaly, Rufin K. Assaré, Fidèle K. Bassa, Naférima Koné, Négnorogo Guindo-Coulibaly, Jan Hattendorf, Jürg Utzinger, Eliézer K. N’Goran

**Affiliations:** 1 Unité de Formation et de Recherche Biosciences, Université Félix Houphouët-Boigny, Abidjan, Côte d’Ivoire; 2 Centre Suisse de Recherches Scientifiques en Côte d’Ivoire, Abidjan, Côte d’Ivoire; 3 Swiss Tropical and Public Health Institute, Basel, Switzerland; 4 University of Basel, Basel, Switzerland; North Carolina State University, UNITED STATES

## Abstract

**Background:**

Preventive chemotherapy using praziquantel is the mainstay for schistosomiasis control. However, there is little evidence on what is supposed to be the most effective school-based treatment strategy to sustain morbidity control. The aim of this study was to compare differences in *Schistosoma mansoni* prevalence and infection intensity between three different schedules of school-based preventive chemotherapy in an area with moderate prevalence of *S*. *mansoni* in Côte d’Ivoire.

**Methodology:**

Seventy-five schools were randomly assigned to one of three intervention arms: (i) annual school-based preventive chemotherapy with praziquantel (40 mg/kg) over four years; (ii) praziquantel treatment only in the first two years, followed by two years whithout treatment; and (iii) praziquantel treatment in years 1 and 3 without treatment in-between. Cross-sectional parasitologic surveys were carried out prior to each round of preventive chemotherapy. The difference in *S*. *mansoni* prevalence and infection intensity was assessed by multiple Kato-Katz thick smears, among children aged 9–12 years at the time of each survey. First-grade children, aged 5–8 years who had never received praziquantel, were also tested at baseline and at the end of the study.

**Principal findings:**

Overall, 7,410 children aged 9–12 years were examined at baseline and 7,223 at the final survey. The baseline prevalence of *S*. *mansoni* was 17.4%, 20.2%, and 25.2% in arms 1, 2, and 3, respectively. In the final year, we observed the lowest prevalence of 10.4% in arm 1, compared to 18.2% in arm 2 and 17.5% in arm 3. The comparison between arms 1 and 2 estimated an odds ratio (OR) of 0.52 but the difference was not statistically significant (95% confidence interval (CI) = 0.23–1.16). Likewise the difference between arms 1 and 3 lacked statistical significance (OR = 0.55, 95% CI = 0.23–1.29). There was no noteworthy difference observed between arms 2 and 3 (OR = 1.06, 95% CI = 0.64–1.75). The lowest *S*. *mansoni* fecal egg counts in the final year survey were observed in arm 1 (7.9 eggs per gram of stool (EPG)). However, compared with 11.5 EPG in arm 2 and 15.4 EPG in arm 3, the difference lacked statistical significance. There were 4,812 first-grade children examined at baseline and 4,513 in the final survey. The overall prevalence of *S*. *mansoni* in these children slightly decreased in arms 1 (from 4.5% to 3.6%) and 2 (from 4.7% to 4.3%), but increased in arm 3 (from 6.8% to 7.9%). However, there was no significant difference in prevalence and infection intensity observed between study arms.

**Conclusions/significance:**

The three treatment schedules investigated led to a reduction in the prevalence and intensity of *S*. *mansoni* infection among children aged 9–12 years. Comparing intervention arms at the end of the study, no statistically significant differences were observed between annual treatement and the other two treatment schedules, neither in reduction of prevalence nor intensity of infection. It is important to combine our results with those of three sister trials conducted simultaneously in other African countries, before final recommendations can be drawn.

## Author summary

The World Health Organization (WHO) recommends preventive chemotherapy with praziquantel as the global strategy for morbidity control of schistosomiasis. The guidelines include target groups and treatment frequencies based on prevalence in school-age children. However, these recommendations are based on expert opinion. The Schistosomiasis Consortium for Operational Research and Evaluation (SCORE) put forward a series of cluster-randomized trials in different African countries, including Côte d’Ivoire, to identify the most suitable approach to gain and sustain the control of schistosomiasis. Results from Côte d’Ivoire did not show statistically significant differences between three school-based treatment schedules (i.e., annual treatment over four years; treatment only in the first two years, followed by two years whithout treatment; and treatment every other year without treatment in-between) in reducing prevalence and intensity of *Schistosoma mansoni* infection among children aged 9–12 years. The results in first-grade children with an age of 5–8 years entering school who had never received deworming drugs showed no significant difference in the prevalence and intensity of *S*. *mansoni* infection between the different treatments at the study end, suggesting that the three strategies were not significantly different for reducing the disease transmission in affected communities. However, our data should be combined with other SCORE studies carried out elsewhere in Africa. A meta-analysis including the results of the sister trials could help to conclude and make more generic recommendations.

## Introduction

Schistosomiasis is a neglected tropical disease (NTD) that remains endemic in sub-Saharan Africa, where it causes a considerable public health burden [1–3]. With the advent of the safe, efficacious, and broad-spectrum antischistosomal drug praziquantel in the late 1970s, the World Health Organization (WHO) endorsed morbidity control as the global strategy [4]. The NTD roadmap, published by WHO in 2012, set targets for 2020, emphasizing preventive chemotherapy, which is the periodic administration of praziquantel to school-age children and other high-risk groups without prior diagnosis [5]. In 2016, 70.9 million school-age children requiring preventive chemotherapy received praziquantel, owing to an estimated coverage of 53.7% [6].

In Côte d’Ivoire, the national schistosomiasis control program was launched in 2012, supported by the Schistosomiasis Control Initiative (SCI). In order to identify the most effective strategy for preventive chemotherapy, taking into consideration operational feasibility and costs, the Schistosomiasis Consortium for Operational Research and Evaluation (SCORE) designed a series of cluster-randomized trials, implemented in several African countries, including Côte d’Ivoire [7–9].

In view of current recommendations for preventive chemotherapy that are largely based on expert opinion, the goal of this study was to generate new evidence about the effectiveness of different school-based treatment strategies for controlling *Schistosoma mansoni* infection. Three school-based treatment strategies were compared in a setting of moderate endemicity, defined as prevalence of infection of 10–24% among children aged 13–14 years, based on duplicate Kato-Katz thick smears. The study endorsed a cluster-randomized design. The primary objective was to assess the difference in prevalence and intensity of *S*. *mansoni* infection between study arms at the final survey with a view to determine which strategy of mass drug administration (MDA) provided the greatest reduction in these indicators in children aged 9–12 years. A secondary objective was to assess the infection prevalence and intensity of first-grade children, aged 5–8 years, in the same population, who entered school and had never received praziquantel, at baseline and at the final survey. This allowed to determine the degree of transmission during the study. Our study complements SCORE trials carried out elsewhere in sub-Saharan Africa [10–12].

## Methods

### Ethics statement

Ethics approval was obtained from the Ministry of Public Health in Côte d’Ivoire (reference no. 1994 MSHP/CNER) and from the Ethics Committee of Basel, Switzerland (reference no. EKBB 279/10). The trial is registered at ISRCTN (reference no. ISRCTN99401114). Local authorities, school directors, teachers, parents, guardians, and children in the study villages were informed about the purpose, procedures, and potential risks and benefits of the study. Parents and guardians of study participants provided written informed consent, while children were assenting. Participation was voluntary, and hence, children could withdraw anytime without further obligations.

### Study area and population

**Fig 1:**
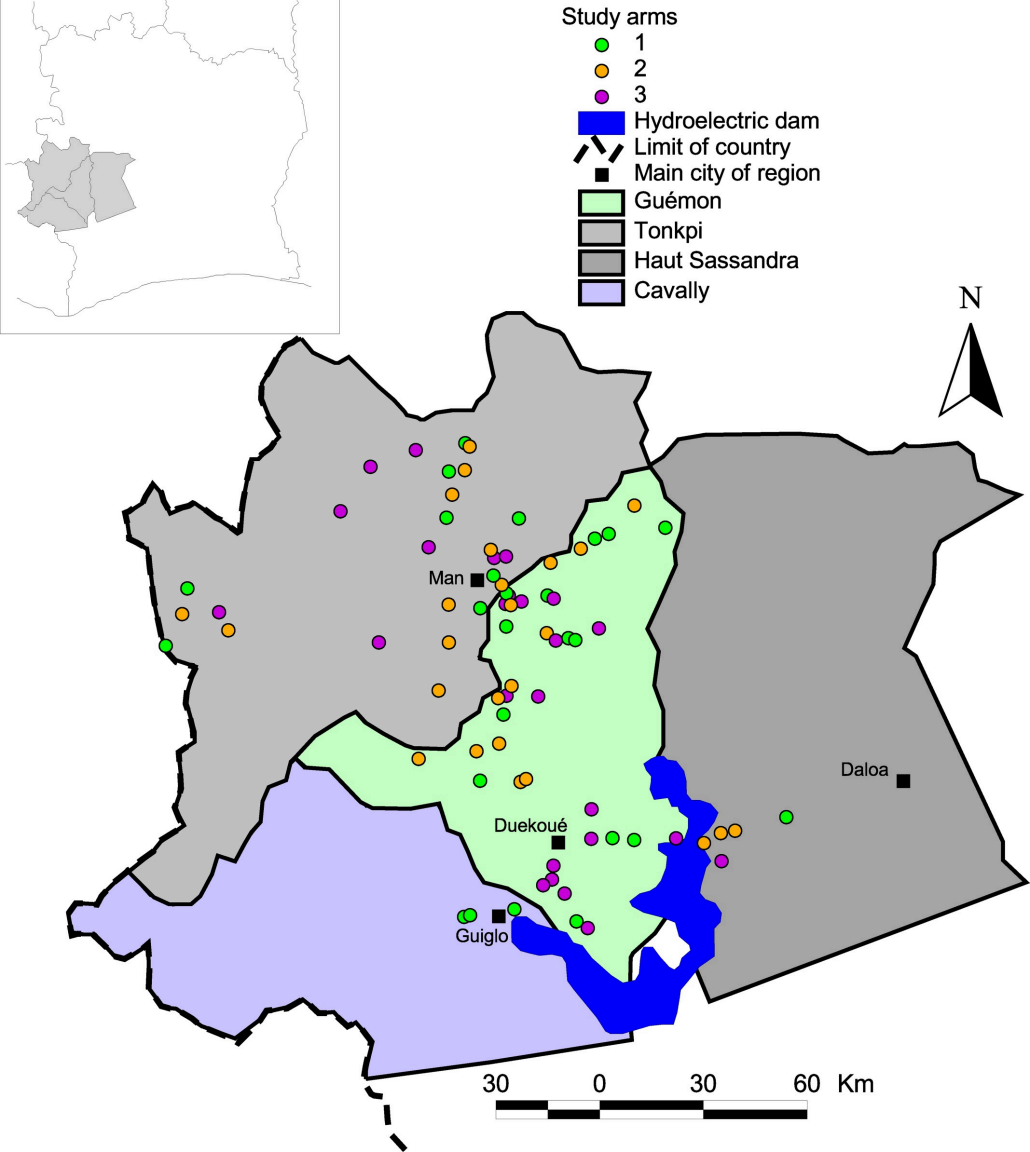
Study area in western Côte d’Ivoire showing the 75 villages, stratified by intervention arms. Arm 1: praziquantel treatment every year; Arm 2: praziquantel treatment only in the first two years, followed by two years whithout treatment; Arm 3: praziquantel treatment every other year without treatment in-between.

The 75 study villages are located in four regions in the western part of Côte d’Ivoire: Cavally, Guémon, Haut-Sassandra, and Tonkpi (Fig 1). This mountainous region with altitudes ranging from 300 m to over 1,000 m above sea level featuring several rivers is known to be endemic for *S*. *mansoni*. The climate is humid tropical with a rainy season from March to October and precipitation varying from 1,200 mm to 2,000 mm per year. The last census of the population in this area, prior to the start of the current study, was conducted in 1998 and revealed approximately 1.5 million inhabitants. The main economic activity in western Côte d’Ivoire is subsistence agriculture [13]. For water supply and other domestic activities, people use rain water, rivers, traditional wells, creeks, fountains, small multi-purpose dams, ponds, tap water, and spring water [14]. Some domestic (e.g., washing dishes and clothes), economic (e.g., fishing), and recreational activities of the communities (e.g., bathing and swimming), are associated with human-water contacts that govern schistosomiasis transmission [15]. School-age children from this region constitute our study population. Of note, this age group is widely recognized as the most exposed to schistosomiasis. In each village, prevalence and intensity of *S*. *mansoni* infection were determined among pupils at school, while all school-age children, whether pupils or not, were taken into account in preventive chemotherapy. Details of the study area and population surveyed have been described elsewhere [16,17].

### Study design and interventions

The study design was a cluster-randomized trial with three parallel arms of 25 villages each. The reason for adopting such a study design was justified on village-level infection dynamics and the fact that preventive chemotherapy is usually school-based. Overall, 75 primary schools were selected and allocated to one of the three arms, using a computer generated block randomized allocation sequence. Each arm was assigned to one particular treatment strategy for a 4-year period. According to the protocol, school-age children in villages of arm 1 received praziquantel treatment every year. Those in villages of arm 2 received treatment only in the first 2 years, followed by two years whithout treatment. Children in villages of arm 3 were treated every other year with no treatment in-between.

Treatment was done through the existing education platform. In a first step, a baseline survey was conducted in each of the 75 villages, followed by four years of interventions according to the different intervention arms. A few days before each assigned treatment, a parasitologic survey was carried out in the study villages among 9- to 12-year-old children. First-grade children, aged 5–8 years, were also tested at baseline and during the final-year survey. The final data collection was implemented in all villages at the end of the 4-year intervention period.

### Eligibility criteria

The selection of study villages has been described elsewhere [9]. In brief, villages were selected for eligibility according to the following criteria: (i) each village has a primary school with at least 100 children aged 9–12 years (determined by readily available school lists); (ii) no recent (within the past 12 months) history of MDA using praziquantel against schistosomiasis, as determined by a questionnaire distributed to health and education personnel; and (iii) prevalence of *S*. *mansoni* ranging between 10% and 24% among a random sample of 50 children aged 13–14 years, as determined by duplicate Kato-Katz thick smears from a single stool sample examined in a rapid appraisal survey.

After a village was deemed eligible, all school-age children, regardless of whether or not they attended school, were included for preventive chemotherapy. However, for parasitologic surveys, only children enrolled in school were included. Children aged 9–12 years with written informed consent signed by parents or guardians in addition to having given oral assent, were randomly selected. At the baseline and final surveys, a sample of first-grade children aged 5–8 years, randomly selected based on the aforementioned eligibility criteria, was included in addition to the 9–12 years age-group in each study arm.

### Parasitologic survey

In each school, 100 randomly selected children aged 9–12 years fulfilling inclusion criteria were provided with empty plastic containers (125 ml). Every morning during three consecutive days, children were invited to provide a small portion of their morning stool in containers. Filled containers were collected, labelled with unique identifiers, and transferred to nearby laboratories in dispensaries and hospitals for diagnostic work-up. Every stool sample was subjected to duplicate Kato-Katz thick smears, using 41.7 mg plastic templates, making two series of slides (A and B) [18]. During the baseline and final surveys, we also recruited up to 100 first-grade children aged 5–8 years per school.

For diagnosis, slides of set A were examined under a microscope by experienced laboratory technicians after a clearing time of 30–45 min. This diagnostic approach provided an opportunity to identify and count hookworm eggs before they cleared and disappeared, along with counting *S*. *mansoni* and other helminth eggs that were recorded separately. Slides of set B were examined a few days later. Approximately 10% of the slides were re-examined by a senior technician for quality control, putting emphasis on *S*. *mansoni*. In case of discrepancies, the slides were re-read until agreement was reached [19,20].

### School-based treatment strategies

Praziquantel was provided by SCI to the national schistosomiasis control program in Côte d’Ivoire, which carried out the preventive chemotherapy supported by staff of the national school health program. First, the regional health authorities were informed about the treatment campaigns. Next, one or two volunteer teachers and one community health worker were trained per village for drug administration. Praziquantel was administered by trained teachers to children with help of the community health workers for the non-enrolled school-age children. Treatment was offered free of charge, with dosing according to WHO guidelines [4]. The coverage rate was calculated by dividing the total number of school-age children treated, regardless of whether they were enrolled or not, by the total number of school-age children in the village, estimated from the total population of each village.

### Statistical analysis

Demographic and laboratory data from the baseline survey were double entered into an Excel spreadsheet (Microsoft Corporation; Redmond, United States of America), and cross-checked with EpiInfo version 3.2 (Centers for Disease Control and Prevention; Atlanta, United States of America). Smartphones were used to directly enter data of all the follow-up surveys. These databases were uploaded and maintained on a central server (Open Data Kit) in Atlanta. A child was considered infected when there was at least one *S*. *mansoni* egg detected on one of the Kato-Katz thick smears. Village level prevalence and study arm level prevalence values were calculated. Reduction in the prevalence of *S*. *mansoni* infection by study arm was calculated using the following formula: prevalence reduction rate = [(prevalence at final survey—prevalence at baseline) / prevalence at baseline] × 100. As not all of the children could provide the three intended stool samples, intensity of infection was determined in eggs per gram of stool (EPG) taking into account the number of samples provided per participant and the fact that each slide contained 41.7 mg of stool. The total number of eggs counted on all the slides of each participant was thus multiplied by a factor of 12, 6, or 4 depending on whether the child provided duplicate, quadruplicate or sextuplicate Kato-Katz thick smears, respectively. Species-specific helminth egg counts were truncated at 1,000 EPG. Children infected with *S*. *mansoni* were classified as light (<100 EPG), moderate (100–399 EPG), and heavy infection (≥400 EPG), according to WHO thresholds.

The arithmetic mean (AM) of infection intensity was evaluated at individual level, excluding negative children, and at village-level, including negative children; both expressed as EPG. The reduction rate in intensity of infection was calculated as follows: egg reduction rate (ERR) = (AM EPG at village level at baseline—AM EPG at village level in the final survey) / AM EPG at village level at baseline x 100. The primary analysis estimated differences between the arms in the final year. Differences in prevalence were assessed using generalized estimating equations (GEE) for binary distributed outcomes with logit link and independent correlation structure to account for potential correlations within village clusters. The adjusted analysis used the same model but included baseline prevalence, sex, and age as additional covariates. Additionally, the adjusted model was weighted to account for the fact that in some villages less than 100 school-age children were sampled. Differences in *S*. *mansoni* egg counts were assessed using GEE for negative binomial distributed outcomes with log link and independent correlation structure. Adjusted and unadjusted models were estimated in the same way as the prevalence models. The same GEE models were also performed by sex to explore the effect of gender on the difference between intervention arms. The primary analysis was done in SAS version 9.4 (SAS Institute Inc.; Cary, United States of America). Details on sample size considerations have been published elsewhere [9]. The statistical analysis plan can be found here [21].

## Results

### Study flow

**Fig 2:**
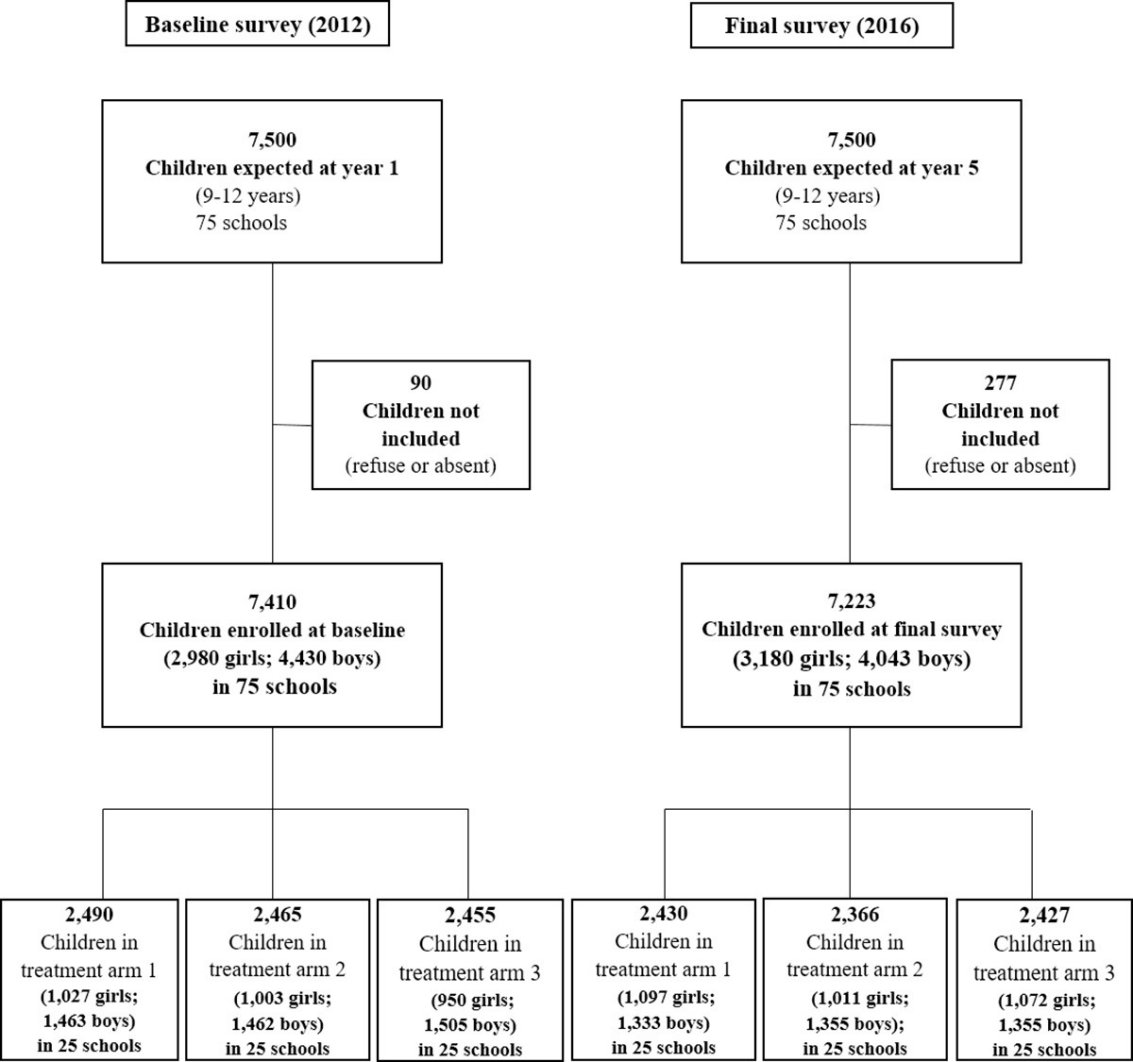
Study profile and number of schools with 9- to 12-year-old participants in each arm. Arm 1: praziquantel treatment every year; Arm 2: praziquantel treatment only in the first two years, followed by two years whithout treatment; Arm 3: praziquantel treatment every other year without treatment in-between.

Parasitologic surveys, interventions, and assessments took place between March 2012 and March 2016. In the first year (2012), 7,500 children aged 9–12 years were randomly selected in the 75 participating schools. Ninety children were missed because of refusal or absence. Hence, 7,410 children were included in the baseline cross-sectional survey consisting of 40.2% of girls with an average age of 10.5 years. The mean number of participants per school was 99.6 in arm 1, 98.6 in arm 2, and 98.2 in arm 3. For the final survey in 2016, again, 7,500 children aged 9–12 years were targeted. Overall, 277 children missed this assessment, resulting in 7,223 children participating. There were 44.0% girls (Fig 2) and the average age was 10.1 years.

In addition, 4,812 first-grade children, aged 5–8 years, were surveyed at baseline and 4,513 in the final survey. The proportion of girls in this age group was 43.2% with an average age of 6.5 years in 2012 and 47.6% with an average age of 6.4 years in 2016.

### Coverage rate of preventive chemotherapy

Preventive chemotherapy was offered only to school-age children. In the first year, coverage of preventive chemotherapy was relatively low in the three arms with 64.2%, 64.4%, and 47.9% in arms 1, 2, and 3, respectively. In subsequent years, the average coverage rate increase to at least 74% in all arms (Table 1). It was observed that in many localities, the coverage rate was below the WHO recommended level of 75%, which was also aimed by the SCORE study protocol [4].

**Table 1:** Coverage of preventive chemotherapy with praziquantel in school-age children by study arm over the four year of intervention in a cluster-randomized trial in Côte d’Ivoire. Arm 1: praziquantel treatment every year; Arm 2: praziquantel treatment only in the first two years, followed by two years whithout treatment; Arm 3: praziquantel treatment every other year without treatment in-between.

Treatment	Study arm	School-age children treated	School-age children total number	% of school-age children treated
Year 1 (2012)	1	8,358	13,010	64.2
	2	11,548	17,937	64.4
	3	6,898	14,413	47.9
Year 2 (2013)	1	10,246	13,755	74.4
	2	15,504	18,534	83.6
Year 3 (2014)	1	13,239	14,231	93.0
	3	12,057	16,232	74.9
Year 4 (2015)	1	14,206	15,778	90.0

### Differences in prevalence and intensity of *S. mansoni* infection in children aged 9–12 years

**Fig 3:**
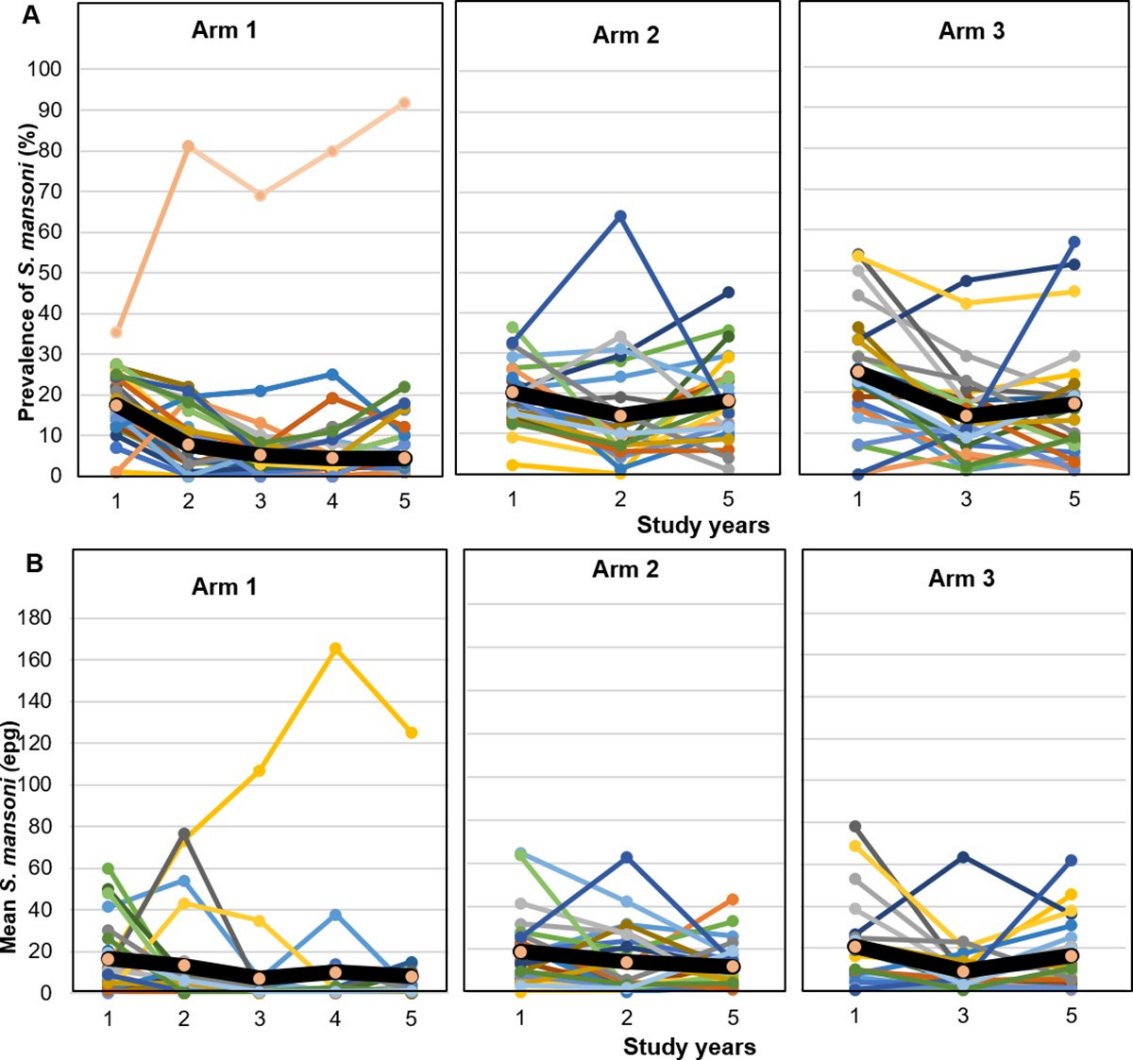
Dynamique of prevalence and infection intensity of *S*. *mansoni* in a 5-year cluster randomized trial among 9- to 12-year-old children in Côte d’Ivoire, stratified by intervention arm. (A): Variation of *S*. *mansoni* prevalence among children aged 9–12 years over time per village and by arm. Each line represents a village and black line represents the overall prevalence of the arm. (B): Variation of arithmetic mean intensity of *S*. *mansoni* per village over year by arm. Each line represents a village and black line represents the overall prevalence of the arm. Arm 1: praziquantel treatment every year; Arm 2: praziquantel treatment only in the first two years, followed by two years whithout treatment; Arm 3: praziquantel treatment every other year without treatment in-between.

The overall *S*. *mansoni* prevalence at baseline was 20.9%. The baseline prevalence in the three treatment arms ranged from 17.4% to 25.2% (Table 2). The final survey showed an overall prevalence of 15.3% in the whole study area among children aged 9–12 years with the lowest prevalence observed in arm 1 (10.4%). However, the estimate was not statistically significantly different from the 18.2% observed in arm 2 (odds ratio (OR) = 0.52, 95% confidence interval (CI) = 0.23–1.16). A similar trend was observed for the difference between arms 1 and 3 (10.4% vs. 17.3%; OR = 0.55, 95% CI = 0.23–1.29). There was also no significant difference between arms 2 and 3 (OR = 1.06, 95% CI = 0.64–1.75) (Table 3). Decreases in pevalence were observed in 22 of 25 schools of arm 1; however, a village named Biélé was characterized as a “persistent hotspot”, where prevalence continued to increase, reaching 91.8% by the study end, despite annual treatment over four years (Fig 3A). A decrease was also observed in 15 and 19 schools in arms 2 and 3, respectively.

**Table 2:** *Schistosoma mansoni* prevalence changes between baseline (2012) and final survey (2016) among children aged 9–12 years in three arms of a cluster-randomized trial in Côte d’Ivoire. Arm 1: praziquantel treatment every year; Arm 2: praziquantel treatment only in the first two years, followed by two years whithout treatment; Arm 3: praziquantel treatment every other year without treatment in-between.

Arm		Baseline (2012)			Final survey (2016)			Absolute change	Relative change (%)
		Examined	Infected	Prevalence (%)	Examined	Infected	Prevalence (%)		
1	Male	1,463	284	19.4	1,333	148	11.1	-8.3	-42.8
	Female	1,027	151	14.7	1,097	104	9.5	-5.2	-35.4
	**Overall**	**2,490**	**435**	**17.5**	**2,430**	**252**	**10.4**	**-7.0**	**-40.2**
2	Male	1,462	328	22.4	1,355	266	19.6	-2.8	-12.5
	Female	1,003	171	17.0	1,011	164	16.2	-0.8	-4.7
	**Overall**	**2,465**	**499**	**20.2**	**2,366**	**430**	**18.2**	**-2.0**	**-9.9**
3	Male	1,505	406	29.9	1,355	261	19.3	-7.6	-28.2
	Female	950	212	22.3	1,072	159	14.8	-7.5	-33.6
	**Overall**	**2,455**	**618**	**25.2**	**2,427**	**420**	**17.3**	**-7.9**	**-31.4**

**Table 3:** Comparison of prevalence and intensity of *S*. *mansoni* infection between arms at the final survey (2016) in a cluster-randomized trial in Côte d’Ivoire. Adjustment of OR and CR are for age, sex, and baseline prevalence. Arm 1: praziquantel treatment every year; Arm 2: praziquantel treatment only in the first two years, followed by two years whithout treatment; Arm 3: praziquantel treatment every other year without treatment in-between.

		Prevalence				Intensity			
Age group	Arms compared	Unadjusted prevalence model estimate OR (95% CI)	P	Adjusted prevalence model estimate OR (95% CI)	P	Unadjusted intensity model estimate CR (95% CI)	P	Adjusted intensity model estimate CR (95% CI)	P
	Arm 1 vs arm 2	0.52 (0.23–1.16)	0.11	0.56 (0.25–1.24)	0.15	0.68 (0.20–2.34)	0.55	0.52 (0.18–1.53)	0.23
9–12 years	Arm 1 vs arm 3	0.55 (0.24–1.30)	0.17	0.72 (0.24–2.14)	0.56	0.51 (0.15–1.77)	0.29	0.39 (0.08–1.93)	0.25
	Arm 2 vs arm 3	1.06 (0.64–1.75)	0.81	1.30 (0.68–2.48)	0.43	0.75 (0.44–1.25)	0.27	0.75 (0.28–2.01)	0.57
	Arm 1 vs arm 2	0.83 (0.23–2.97)	0.77	0.80 (0.30–2.11)	0.65	0.64 (0.14–2.97)	0.57	0.62 (0.13–3–03)	0.56
5–8 years	Arm 1 vs arm 3	0.47 (0.13–1.72)	0.25	0.73 (0.19–2.82)	0.65	0.37 (0.08–1.71)	0.20	0.40 (0.08–1.97)	0.26
	Arm 2 vs arm 3	0.57 (0.31–1.05)	0.07	0.92 (0.44–1.89)	0.81	0.58 (0.23–1.42)	0.23	0.65 (0.29–1.45)	0.29

**CI**: confidence interval; **CR**: count ratio; **OR**: odds ratio

**Fig 4:**
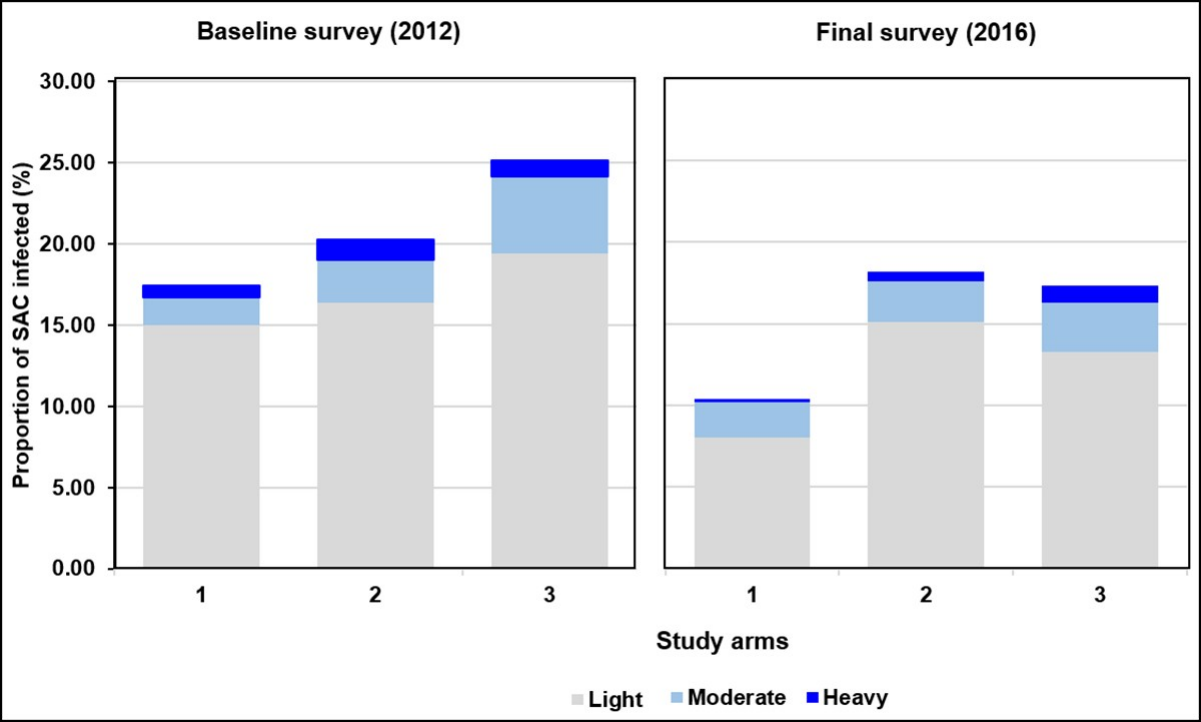
Variation of proportion in each infection status category of *S*. *mansoni* among 9- to 12-year-old children per arm from baseline (2012) to final survey (2016). SAC: school-age children; Arm 1: praziquantel treatment every year; Arm 2: praziquantel treatment only in the first two years, followed by two years whithout treatment; Arm 3: praziquantel treatment every other year without treatment in-between.

The mean infection intensity by arm at baseline (including egg-negative children) were 12.4 EPG, 17.4 EPG, and 20.1 EPG in arms 1, 2, and 3, respectively. Similarly to the prevalence, infection intensity was lowest in arm 1 in the final year with a mean *S*. *mansoni* egg count of 7.9 EPG compared with 11.5 EPG in arm 2 and 15.4 EPG in arm 3 (Table 4). However, also in this case, the CIs were large and included unity (Table 3). Adjusting for baseline prevalence, age, and sex did not noteworthy change the estimates. The variation in this AM intensity of infection per school per arm showed an almost continuous decline over time in arms 1 and 2. However, in arm 3, a rapid increase in this mean intensity of infection was observed (Fig 3B). In the final survey, it was noted that the proportion of heavy infections (≥400 EPG) decreased from 0.8% to 0.2% in arm 1 and from 1.2% to 0.6% in arm 2, but remained stable in arm 3 (1.0%) (Fig 4). No significant differences related to sex were observed in the study outcomes.

**Table 4:** *Schistosoma mansoni* intensity changes between baseline (2012) and final survey (2016) among 9- to 12-year-old children in three arms of a cluster-randomized trial in Côte d’Ivoire. Arm 1: praziquantel treatment every year; Arm 2: praziquantel treatment only in the first two years, followed by two years whithout treatment; Arm 3: praziquantel treatment every other year without treatment in-between.

Arm		Baseline (2012)		Final survey (2016)		Egg reduction rate
		Individual-level AM infection	Village-level AM infection	Individual-level AM infection	Village-level AM infection	
1	Male	77.1	15.2	89.8	9.8	35.5%
	Female	59.8	8.7	58.0	5.6	35.7%
	**Overall**	**71.1**	**12.4**	**76.7**	**7.9**	**36.0%**
2	Male	108.1	24.5	72.1	14.5	41.0%
	Female	49.7	7.9	50.5	8.1	-2.0%
	**Overall**	**88.1**	**17.4**	**63.8**	**11.5**	**33.8%**
3	Male	88.1	23.5	97.7	18.8	20.0%
	Female	66.9	14.7	77.0	11.2	24.1%
	**Overall**	**80.8**	**20.1**	**89.9**	**15.4**	**23.1%**

**AM:** arithmetic mean; **EPG**: eggs per gram of stool; **Individual-level AM infection**: excluding negative children expressed as EPG; **Village-level AM infection:** including negative children expressed as EPG

### Differences in prevalence and intensity of *S. mansoni* infection among first-grade children

A total of 4,812 first-grade children entering school (before receiving preventive chemotherapy) participated in the baseline survey, of whom 257 (5.3%) were infected with *S*. *mansoni*. At the final survey, among the 4,513 participating first-grade children, the prevalence decreased slightly from 4.5% to 3.6% in arm 1 and from 4.7% to 4.3% in arm 2, but slightly increased in arm 3 from 6.8% to 7.9%. Means of infection intensity at village level also varied in the same order across study arms (Table 5). In this age group, no significant difference was observed between the study arms in reduction of prevalence and infection intensity at the study end (Table 3).

**Table 5:** Prevalence and intensity of *S*. *mansoni* infection among 5- to 8-year-old children for baseline (2012) and final survey (2016) in a cluster-randomized trial in Côte d’Ivoire. Arm 1: praziquantel treatment every year; Arm 2: praziquantel treatment only in the first two years, followed by two years whithout treatment; Arm 3: praziquantel treatment every other year without treatment in-between.

Study year	Study arm	Number tested	Number infected	Prevalence (%)	Heavily infected (%)	Individual-level AM infection	Village-level AM infection
Baseline (2012)	1	1,409	63	4.5	9 (0.6)	165.9	8.2
	2	1,750	82	4.7	9 (0.5)	143.7	5.8
	3	1,653	112	6.8	9 (0.5)	129.7	8.6
	**Total**	**4,812**	**257**	**5.3**	**27 (0.6)**	**146.4**	**7.5**
Final survey (2016)	1	1,442	52	3.6	1 (0.1)	83.6	2.8
	2	1,499	65	4.3	5 (0.3)	108.6	4.7
	3	1,572	116	7.4	10 (0.6)	110.8	7.4
	**Total**	**4,513**	**233**	**5.2**	**16 (0.4)**	**101.0**	**4.9**

**AM**: arithmetic mean; **Individual-level AM infection**: excluding negative children expressed as EPG; **Village-level AM infection:** including negative children expressed as EPG; **EPG**: eggs per gram of stool

## Discussion

Preventive chemotherapy with praziquantel is the key strategy for morbidity control of schistosomiasis, as recommended by WHO and implemented by national control programs [6]. National control programs need reliable data to monitor the effectiveness of currently recommended policies, in order to refine and enhance strategies should drug interventions fail. The current recommendation for preventive chemotherapy with praziquantel in moderate endemicity areas is to treat school-age children once every other year [22]. In our study, in addition to this recommended approach, we also tested two additional treatment schedules: annual treatment and treatment on two consecutive years, followed by two years without treatment. The effectiveness of the treatment strategy was evaluated by key indicators, including the prevalence and intensity of *S*. *mansoni* infection in the targeted group (children aged 9–12 years) and among first-grade children (aged 5–8 years) entering school. Our study compared the three treatment strategies by monitoring these primary outcomes over a 5-year period. As expected, data confirmed that the three treatment strategies all yielded reductions in the overall prevalence of *S*. *mansoni* infection in school-age children. However, in the final survey, no statistically significant difference in prevalence and intensity of *S*. *mansoni* infection was observed between study arms. Our results corroborate findings from other SCORE studies conducted elsewhere in sub-Saharan Africa [10,11,23,24]. Additionally, there was no significant difference in prevalence and intensity of infection between study arms at the final survey among first-grade children who have never been treated before, suggesting a similar impact on the *S*. *mansoni* transmission in the community regardless of the treatment schedule.

Despite the reductions in prevalence observed at the study end, all the study arms remained in the moderate endemicity category, as defined by WHO (prevalence between 10% and 49%) and several cases of heavy intensity infections have been observed even among children aged 5–8 years. Hence, it seems difficult that a control program emphasizing preventive chemotherapy alone will reach the goal of eliminating schistosomiasis as a public health problem.

There was one village, Biélé, which is considered as a persistent hotspot [25–27] where the force of transmission was apparently very high, heavily influencing the overall prevalence in arm 1. Despite the fact that coverage of preventive chemotherapy in this village increased over the four years of intervention to reach 75% in the final year, the prevalence increased gradually up to a very high level (92%) at the end of the study. In such settings, extending preventive chemotherapy to the whole population, increasing the frequency of treatments, or supplementing other interventions (e.g., snail control and behavioral changes to reduce human-water contact) as previously suggested [28–30], are warranted.

The relatively low coverage for preventive chemotherapy compared to the standard recommended by WHO, observed in the three study arms during the first year was, at least partially, explained by a post-election crisis, which affected the stability of the education system and community cohesion in the area, and limited our efforts for population sensitization [31]. This was the main limitation of the study. Following significant improvements in the political situation after 2013, the school-based treatment achieved an average coverage rate of at least 74% in all arms in subsequent years. This rate is close to the minimal target (75%) recommended by WHO [22] and the SCORE protocol and should instead contribute to further reduce prevalence and intensity. Taking into account the fact that the coverage targeted could not be reached in several villages of the study and that treatment coverage was an intermediate variable on the causal path between treatment and prevalence, we did not include coverage in the model as done for another SCORE study [11]. However, high treatment coverage rates from the first year of intervention could have resulted in better reduction of prevalence and intensity of *S*. *mansoni* in the study arms.

In conclusion, our results showed that the three school-based treatment strategies with praziquantel investigated, achieved reduction in prevalence and intensity of *S*. *mansoni* infection among children aged 9–12 years. At the end of the study, no statistically significant differences were observed between the study arms. However, it is important to put our results in context with findings derived from three sister trials conducted simultaneously in other countries, before final recommendations can be drawn. In view of the relatively modest reductions in prevalence and infection intensity observed in all the three study arms, preventive chemotherapy alone was insufficient to consider the ultimate goal of schistosomiasis elimination [12].
